# Investigating the Effect of Microwave Induction on the Polymerization Rate of Polycarboxylate Superplasticizers

**DOI:** 10.3390/polym16030322

**Published:** 2024-01-24

**Authors:** Liran Zhang, Wenqian Du, Dongmin Wang, Yue Zhang, Fang Wang, Dawang Zhang, Yang Chen, Xinyue Zhai, Yingchun Liu, Xiao Yi

**Affiliations:** 1 School of Materials Design and Engineering, Beijing Institute of Fashion and Technology, Beijing 100029, China; 20180022@bift.edu.cn (L.Z.); duwenqian2022@email.szu.edu.cn (W.D.); 19967291321@163.com (Y.C.); 17736265350@163.com (X.Z.); facailiu5520@outlook.com (Y.L.); yixiao123467958@163.com (X.Y.); 2 Department of Chemical Engineering, China University of Mining & Technology, Beijing 100083, China; wangfang_2020@zzu.edu.cn (F.W.); zhangdawang@xauat.edu.cn (D.Z.); 3 School of Civil and Transportation Engineering, Shenzhen University, Shenzhen 518060, China; 4 School of Civil Engineering, Qingdao University of Technology, Qingdao 266520, China

**Keywords:** PCE, microwave induction, polymerization kinetics, thermal effect, permittivity

## Abstract

As a transmission medium and heating energy, microwave is widely favored due to its high efficiency, strong selectivity, and easy control. Here, the effects of different heating methods (conventional thermal induction (CI) and microwave induction (MI)) on the polymerization rate of polycarboxylate superplasticizer (PCE) were investigated. Compared with CI, MI significantly boosted the polymerization rate (by approximately 51 times) and markedly decreased the activation energy (*Ea*), from 46.83 kJ mol^−1^ to 35.07 kJ mol^−1^. The polar of the monomers and initiators in the PCE synthesis contributes to varying permittivities and loss factors under the microwave field, which are influenced by their concentration and reaction temperature. The insights gained from the microwave thermal effects and the micro-kinetics of the PCE polymerization system are able to propose theoretical underpinnings for the industrial-scale application of microwave induction polymerization, potentially steering the synthesis of polymer materials towards a more efficient and cleaner process.

## 1. Introduction

Superplasticizers (SPs), essential components in modern concrete, can optimize the microstructure of fresh concrete and enhance its workability [1,2,3,4]. Among these, polycarboxylate superplasticizers (PCEs) are particularly noteworthy for their high efficiency water-reducing properties due to their high water-reducing rate and easy modification of polymer structure to gain target performance [5,6,7]. However, current production technology of PCE face challenges including complex processing methods, prolonged production time, high energy consumption, and unscreened raw materials, which limit its development and application. Developing simple processes, utilizing eco-friendly raw materials and products, and ensuring superior performance are critical for energy conservation, environmental protection and sustainable development of PCE and other related copolymer production processes and technologies [8]. 

Currently, conventional thermal induction (CI) remains the major approach for PCE copolymer synthesis worldwide [9,10,11,12]. Compared to CI with heat exchange through water/oil heating, microwave, as a transmission medium and heating energy, is gaining popularity [13,14,15,16,17], because of its advantages of cleanliness, environmental protection, precise target, and ease of control. Recent studies, along with previous work of our research group [18,19,20,21], indicates that PCE synthesized by microwave induction (MI) exhibits excellent dispersion performance, and the monomer ratio in the copolymer is closer to the intended design. Compared with thermal conduction induction (e.g., water bath heating), the reaction time of PCE synthesis by MI is reduced by eight times and the conversion rate is increased by 26.1%. The microwave-induced synthesis process not only takes less time and energy but also optimizes the molecular structure and properties of copolymers. The main advantage of MI over CI lies in the microwave directly to the dipole or ionic molecule present in the reaction mixture, and the energy transfer occurs in less than a nanosecond (10^−9^ s), ultimately leading to a rapid rise in temperature. In contrast, CI relies on slower and less efficient heat conduction through container walls to the solvent and reactants [22]. This means that the reaction rate of microwave heating is much higher than that of traditional water bath heating. Therefore, the reaction rate under MI substantially exceeds that of CI. However, a clear and systematic experimental foundation to analyze the kinetics and mechanism of microwave-induced polymerization is lacking. The application of microwave technology in the chemical industry is a general trend, but one of the main obstacles preventing its effective application is temperature calibration and control in polymer chemistry and processing. The problems in the process of industrialization cannot be solved by theoretical research, which limits the application and development of microwave technology.

In polymer chemistry, it is understood that the polymerization rate correlates with the conversion rate of the reaction. Methods to determine conversion rate are broadly classified into direct and indirect approaches. Direct methods, such as the commonly used precipitation technique, involve polymerizing reactants at a specific temperature and taking regular samples to measure the amount of polymer formed at different intervals. Indirect methods assess changes in physical and chemical properties of the polymerization system, such as specific volume, viscosity, refractive index, and absorption spectrum. These properties are used to calculate the decrease in monomer concentration or the increase in polymer quantity. Standard indirect methods include the weighing method, refractive index method, dielectric constant method, and the swelling agent method. Among these, the swelling agent method is notably the simplest for determining reaction conversion.

In this study, we utilized the dilatometer method to measure the polymerization conversion, comparing the polymerization rates under MI and CI. At the same time, the thermal effects induced by microwaves were explored through the permittivity of the reaction medium. Based on this, the mechanism of microwave-induced polymerization rate was analyzed, which provided a basis for the application of microwave energy in PCE industrial preparation.

## 2. Materials and Methods

### 2.1. Raw Materials

α-methally-ω-hydroxy poly (ethylene glycol) ether (HPEG) with Mw of ca. 2400 was provided by Liaoning Oxiranchem, Inc. (Liaoyang, China). Analytical grade chemicals, acrylic acid (AA), hydrogen peroxide (H_2_O_2_) (ca. 30% purity), 3-mercaptopropionic acid (MPA), and ascorbic acid (VC) (all ≥ 98% purity) were purchased from Shanghai Aladdin Biochemical Technology Co., Ltd. (Shanghai, China), and were used as received. Deionized (DI) water was used in all experiments.

### 2.2. Determination of Kinetic Parameters of Polymerization

Polymerization kinetics primarily investigates the quantitative relationship between reaction rate and monomer concentration, initiator concentration and temperature. Typically, the polymerization rate is measured in terms of monomer consumption or polymer production per unit time. However, conversion time data are the most fundamental experimental measurements. In a polymerization reaction, the density of the unreacted monomer is relatively low compared to the higher density of the polymer. As the reaction progresses, the polymer content increases, leading to a gradual decrease in the total volume of the polymerization system. The degree of volume shrinkage is proportional to the conversion of the monomer. In this study, the dilatometer method [23] was employed to measure the volume shrinkage value in the polymerization process and calculate the conversion rate. To improve the accuracy and sensitivity of measuring volumetric shrinkage, a narrow diameter capillary tube was attached to the top of the dilatometer. System volume changes were directly observed through the movement of the liquid level in the capillary tube. The conversion was determined as outlined in Formulas (1) and (2) below.

(1) $$C_{V}=\frac{∆V}{KV_{0}}\times 100\%$$

(2) $$∆V=\pi r^{2}∆h$$

where, $C_{V}$ is the conversion rate. ∆V represents the volume shrinkage at time *t* of the polymerization reaction. $V_{0}$ represents the initial volume of polymerization. ∆h is the drop height of the dilatometer capillary and r is the radius of the capillary. K is the volume contraction factor, which is determined by the residual double bond content in the polymerization by the titrations with potassium bromate and potassium bromide. The specific determination method is as follows: A certain concentration of monomer and initiator aqueous solution is prepared and placed into the ampere bottle of the dilatometer, and the capillary is quickly tilted and inserted into the microwave reactor (XH-100B, Beijing XiangHu Science and Technology Development Co., Ltd., Beijing, China) or thermostat water bath (DF-101S, Zhejiang Lichen Instrument Technology Co., Ltd., Shaoxing, China) for temperature control. In the MI method, the output reactor’s power is adjusted, and its temperature is finely controlled by a contact temperature sensor (temperature accuracy of ±1 °C) complemented by an air-cooling system. It is equipped with an electromagnetic stirring system to guarantee a uniform reaction temperature. In the CI method, a 525 W digital display water bath capable of maintaining a stable temperature water bath is used, and the temperature accuracy is ±1 °C.

$V_{0}$ denotes the capillary volume when the liquid level rises to its highest point. Upon observing a decrease in the capillary’s liquid level to a predetermined height, the dilatometer should be promptly removed. ∆V can be calculated using Formula (2). Then, a precise amount of reaction liquid is taken into the iodine measuring bottle, and excessive KBr-KBrO_3_ solution and a certain amount of hydrochloric acid solution are added. KBr reacts with KBrO_3_ under acidic conditions to form Br_2_. After the completion of the addition reaction, any unreacted bromine is determined by iodometry. K can be obtained by calculating the monomer content in the product through Formula (1). Reference GB/T 601-2002 Chemical reagent—Preparations of standard volumetric solutions describes the preparation of the titration solution used in this experiment [24].

It is worth noting that the dilatometer method is more accurate only when the conversion rate is in the range of 10%. When the conversion rate is more than 10%, the viscosity of the polymerization reaction system increases, and the data measured by the dilatometer are no longer the real conversion rate of the system. Polymerization rate (*Rp*) is generally quantified as the amount of polymer produced per unit time or the consumption of monomer, so it can be obtained from the slope of the conversion-time ($C_{V}-t)$ curve. Equation (3) below delineates the computation of *Rp*.

(3) $$R_{P}=-\frac{d[M]}{dt}=[M]\frac{dC_{V}}{dt}$$

In the microscopic kinetics of radical polymerization, the kinetic equation [25] can be rewritten as:

(4) $$R_{P}=kC_{M}^{m}C_{I}^{n}$$

where k is the polymerization rate constant. $C_{M}$ and $C_{I}$ are monomer and initiator concentrations, respectively.

To compare the effects of the MI and CI methods on the synthesis of PCE, the activation energy (*Ea*) and preexponential factor (*A*) of the reaction are calculated using the Arrhenius equation (Equation (5) below) related to the reaction rate constant [26].

(5) $$k=A\cdot e^{-\frac{E_{a}}{RT}}$$

Combining with the Equation (4), the kinetic equation of polymerization reaction can be written as Equations (6) and (7) below.

(6) $$R_{P}=Ae^{-\frac{E_{a}}{RT}}\cdot C_{M}^{m}C_{I}^{n}$$

(7) $${lnR}_{p}=-\frac{E_{a}}{R}\cdot \frac{1}{T}+C$$

### 2.3. Determination of Permittivity

The measurement of permittivity is critical for understanding the interaction between the reaction medium and microwaves during the polymerization of PCE. At the macroscopic level, the conversion of absorbed microwaves into heat in a dielectric (i.e., nonconductive) material is described through the dielectric continuum model, so the permittivity of a material can be expressed as Equation (8) below [27].

(8) $$\varepsilon =\varepsilon^{\prime }+{i\varepsilon }^{\prime \prime }$$

where $\varepsilon^{\prime }$ is the real component of the permittivity, which describes the constant of the polarizability of the dielectric molecule in the electric field. $\varepsilon^{\prime \prime }$ is the imaginary component of the permittivity, which represents the efficiency with which the medium converts electromagnetic waves into heat.

The relationship between relative permittivity and temperature can be well described by an exponential function (Equation (9) below) like the Arrhenius equation [28].

(9) $$\varepsilon =A\cdot e^{\frac{E^{*}}{k_{B}T}}$$

The parameter $E^{*}$ is defined as the energy barrier of overcoming dipole–dipole interactions between molecules herein, which can indicate the sensitivity of the ε of a solvent to change with temperature.

Energy dissipation in a dielectric medium is often quantified by the loss tangent (tan δ), which can be obtained from permittivity via the ratio of loss over storage component according to Equation (10) below.

(10) $$tan\;\delta =\frac{\varepsilon^{\prime \prime }}{\varepsilon^{\prime }}$$

tan δ represents the ability to convert electromagnetic energy into thermal energy at specific frequencies and temperatures. The reaction medium with a higher tan δ value can absorb microwave energy more efficiently and be heated rapidly in the microwave field. In this experiment, the permittivity of the reaction solution was determined by the apparatus of Kama Huang’s research group [29,30,31].

### 2.4. Simulation and Calculation of Electrostatic Potential and Dipole Moment

In this study, all the quantum chemistry calculations of the four molecules were conducted by the Gaussian 09 package. The configuration, electronic information and chemical properties of compounds can be described by performing geometrical relaxation and single-point calculations. The geometric optimization and the single-point calculation of the target material were carried out at the B3LYP/ 6-311G (d,p) level. To involve Van der Waals action, a DFTD3(BJ) correction was added to the calculation. The three-dimensional visualization was realized by VMD software (1.9.4).

## 3. Results and Discussion

### 3.1. Microscopic Polymerization Kinetics Analysis of PCE under Different Heating Methods

In this study, the effects of the MI and CI methods on the polymerization conversion rate of PCE were compared. The effects of total monomer concentration (*C_M_*), total initiator concentration (*C_I_*), microwave power and polymerization temperature on conversion rate (*C_V_*) and polymerization rate (*R_p_*) were evaluated. Considering that the chain transfer agent only reduced the degree of polymerization and did not affect the polymerization rate, the effect of chain transfer agent was not evaluated.

#### 3.1.1. The Effects of *C_M_* on *C_V_* and *R_p_*

The PCE synthesized is a binary copolymer, and the experiment focused on the effects of *C_M_*, with molar ratios *n_AA_*:*n_HPEG_* = 2.5:1, 3:1, 4:1, and 5:1 on the polymerization kinetics. The impacts of *C_M_* (1.0, 1.2, 1.5, 1.8, and 2.1 mol·L^−1^) were investigated, while the other factors remained unchanged; the concentration of chain transfer agent (*C_T_*) was 0.016 mol·L^−1^, *C_I_* fixed at 0.055 mol·L^−1^ and *n*_*H*_2_*O*_2__: *n_VC_* was 5.85:1, the polymerization reactions were carried out at 50 °C and under a microwave power of 600 W.

**Figure 1:**
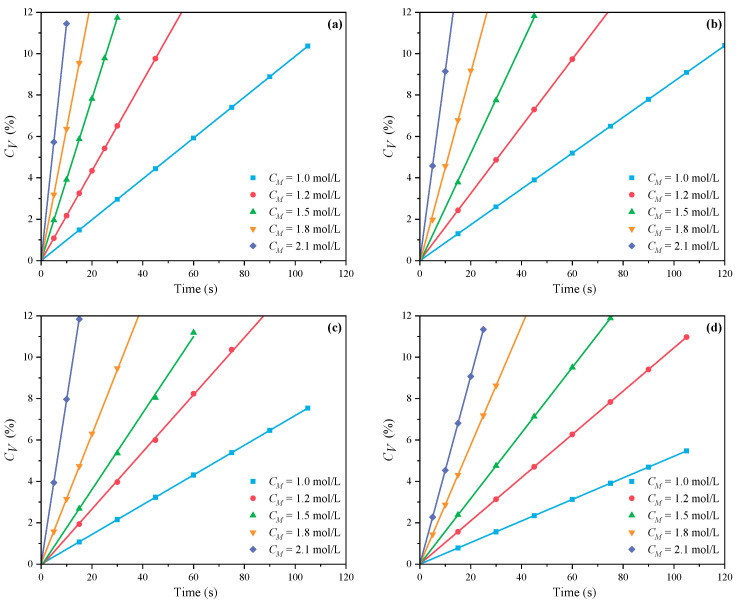
Effects of *C_M_* on *C_V_* at *n_AA_*:*n_HPEG_* of (**a**) 2.5:1, (**b**) 3:1, (**c**) 4:1, and (**d**) 5:1 under MI.

**Figure 2:**
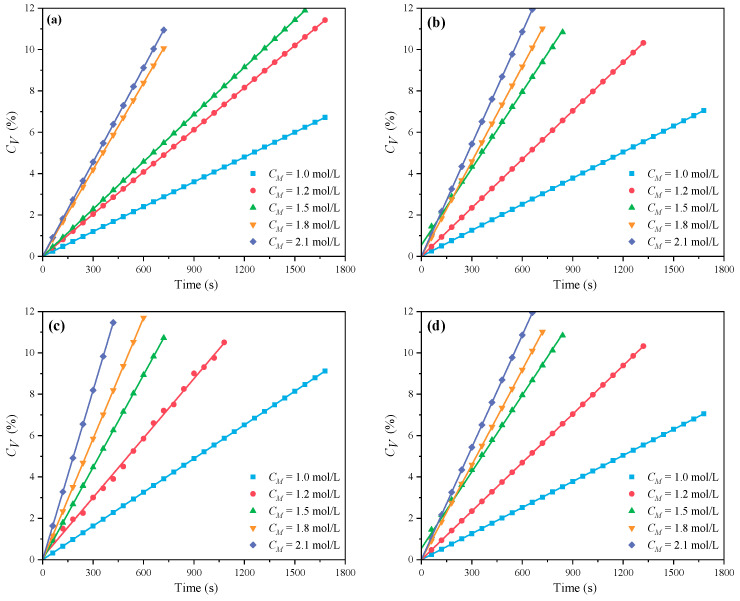
Effects of *C_M_* on *C_V_* at *n_AA_*:*n_HPEG_* of (**a**) 2.5:1, (**b**) 3:1, (**c**) 4:1, and (**d**) 5:1 under CI.

Figure 1 and Figure 2 demonstrate the outcomes of the MI and CI methods, respectively. The *C_V_* displays an increasing trend with the increase of *C_M_* at *n_AA_*:*n_HPEG_* = 2.5:1, 3:1, 4:1, and 5:1. Because there were fewer excitable monomers and a reduced chance of monomer collisions with live radicals, a reduced *C_M_* correlated with a lower *C_V_*, which consequently decreased the initiation efficiency and the polymerization rate *R_p_*. In contrast, an increase in *C_M_* promoted more frequent collisions between monomers and radicals, thus accelerating the polymerization process and enhancing *C_V_*.

**Figure 3:**
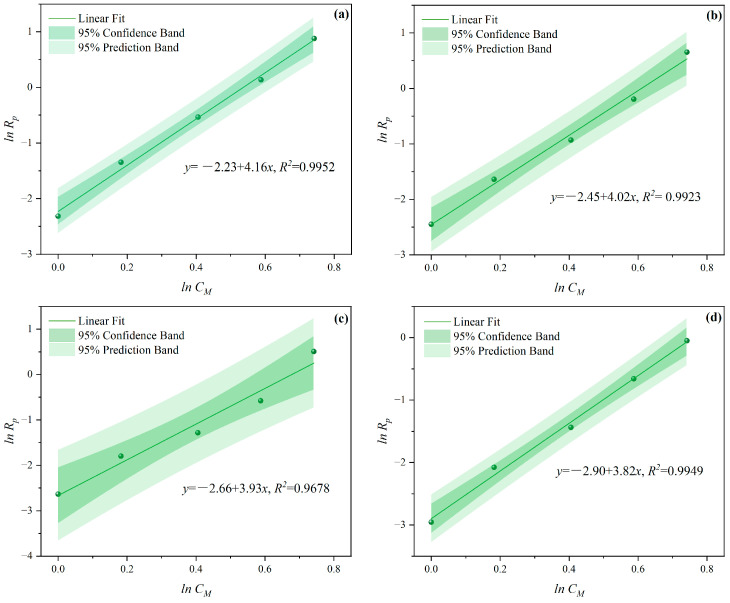
Effects of *C_M_* on *R_p_* at *n_AA_*:*n_HPEG_* of (**a**) 2.5:1, (**b**) 3:1, (**c**) 4:1, and (**d**) 5:1 under MI.

**Figure 4:**
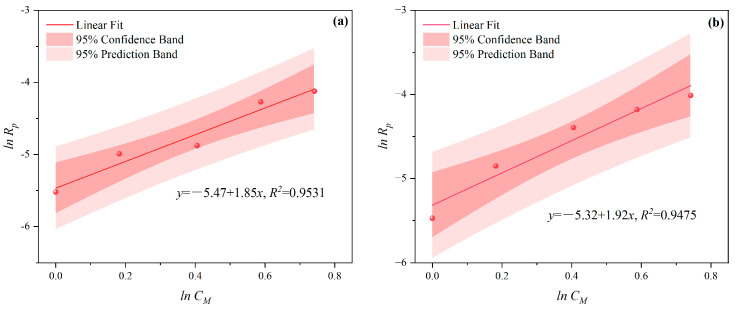

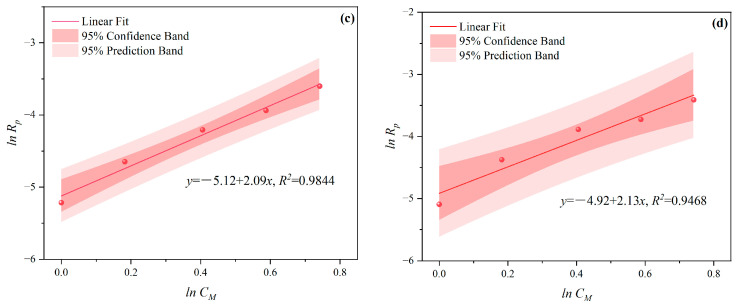
Effects of *C_M_* on *R_p_* at *n_AA_*:*n_HPEG_* of (**a**) 2.5:1, (**b**) 3:1, (**c**) 4:1, and (**d**) 5:1 under CI.

It was found that $lnC_{M}$ and $lnR_{P}$ showed a good linear relationship, as illustrated in Figure 3 and Figure 4. In these figures, confidence band and prediction band are introduced, the former indicating the fitting degree between polymerization rate and various influencing factors, and the latter representing a range not limited to X; we can predict its polymerization rate. The slopes of MI polymerization all exceeded 3, and are shown as 4.157 in Figure 3a, 4.024 in Figure 3b, 3.928 in Figure 3c, and 3.816 in Figure 3d. These results indicated the relationships *R_p_∝CM*^4.157^, *R_p_∝CM*^4.024^, *R_p_∝CM*^3.928^, and *R_p_∝CM*^3.816^, respectively. The slopes for the CI method were slightly lower, with values of 1.856 in Figure 4a, 1.916 in Figure 4b, 2.088 in Figure 4c, and 2.133 in Figure 4d, reflecting the relationships *R_p_∝C_M_*^1.856^, *R_p_∝C_M_* ^1.916^, *R_p_∝C_M_*^2.088^, and *R_p_∝C_M_*^2.133^, respectively.

Previous studies [21,32] found that the relative activities of the two monomers under MI and CI were different. Specifically, MI significantly enhanced the relative activity of HPEG macromonomer, while AA demonstrated a higher relative activity under CI. Therefore, an appropriate low *n_AA_*:*n_HPEG_* was necessary to enhance the *C_V_* under the MI method. Thus, to improve the conversion rate *C_V_* with MI, a lower ratio of *n_AA_*:*n_HPEG_* was optimal, whereas CI benefitted from a higher ratio for effective polymerization. The influence of MI on monomer activity was attributed to the interaction of their polarity with the microwave electromagnetic field; a detailed mechanism of this will be presented in Section 3.2.

**Figure 5:**
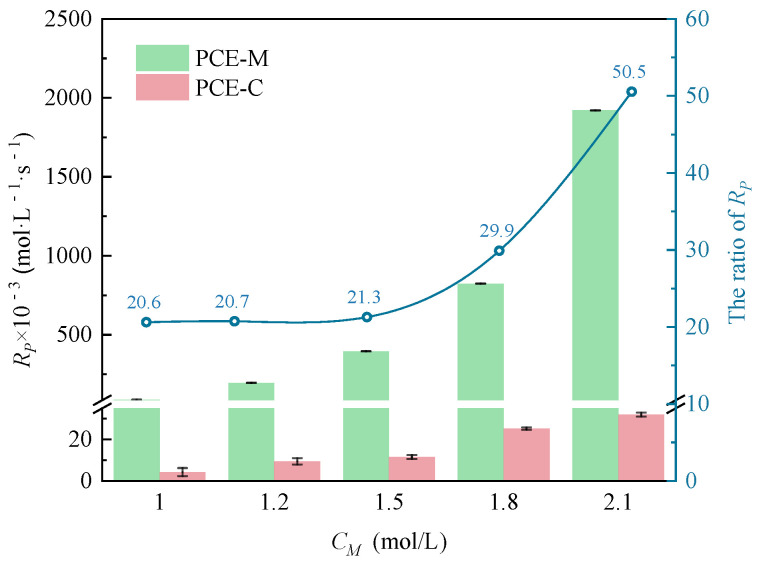
Comparison of *C_M_* on *R_p_* at *n_AA_*:*n_HPEG_* of 3:1 under two different heating methods.

Based on this ratio between the monomers, further evaluation and comparison of *R_p_* was made under MI or CI, as shown in Figure 5. It was observed that with the increase of *C_M_*, *R_p_* under both MI and CI increased gradually. At each *C_M_* (1.0, 1.2, 1.5, 1.8, and 2.0 mol·L^−1^), *R_p_* under MI was significantly higher than that under CI, and the advantage of the MI became more and more obvious. *R_p_* was found to be 20.61~50.54 times higher under MI than under CI. This considerable difference underscored the enhanced likelihood of collisions between monomers and free radicals under MI, which was a contributing factor to the substantially elevated *R_p_*.

#### 3.1.2. The Effects of C_I_ on C_V_ and R_p_

**Figure 6:**
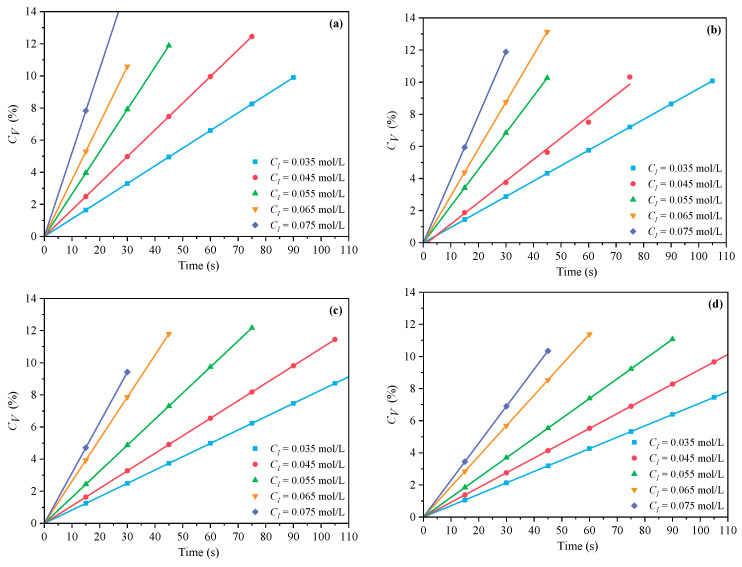
Effects of *C_I_* on *C_V_* at *n*_*H*_2_*O*_2__:*n_VC_* of (**a**) 3.5:1, (**b**) 4.65:1, (**c**) 5.85:1, and (**d**) 7:1 under MI.

**Figure 7:**
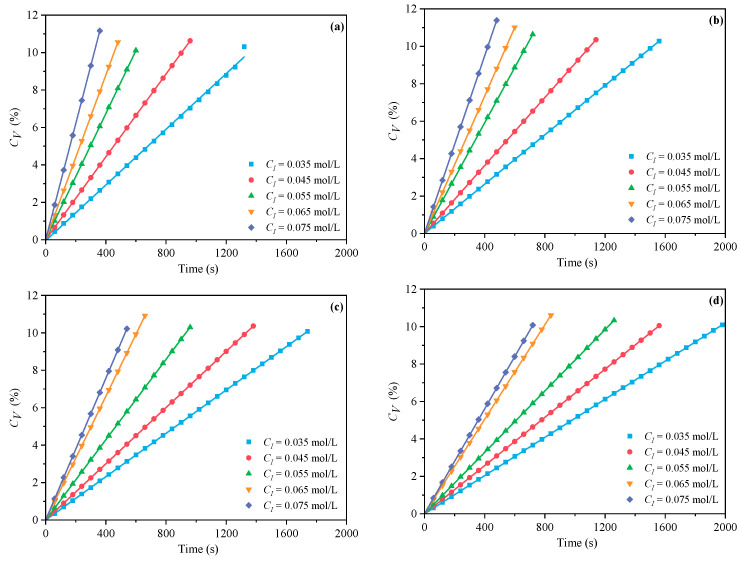
Effects of *C_I_* on *C_V_* at *n*_*H*_2_*O*_2__:*n_VC_* of (**a**) 3.5:1, (**b**) 4.65:1, (**c**) 5.85:1, and (**d**) 7:1 under CI.

**Figure 8:**
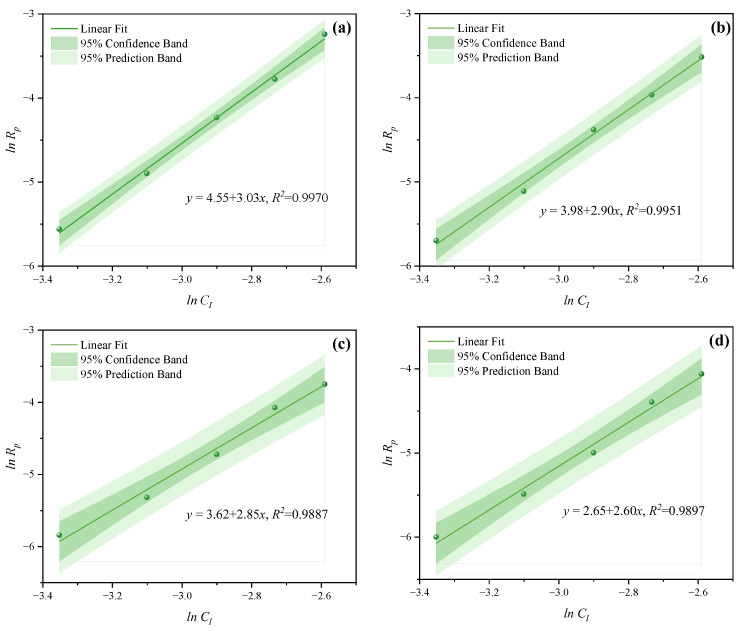
Effects of *C_I_* on *R_p_* at *n*_*H*_2_*O*_2__:*n_VC_* of (**a**) 3.5:1, (**b**) 4.65:1, (**c**) 5.85:1, and (**d**) 7:1 under MI.

**Figure 9:**
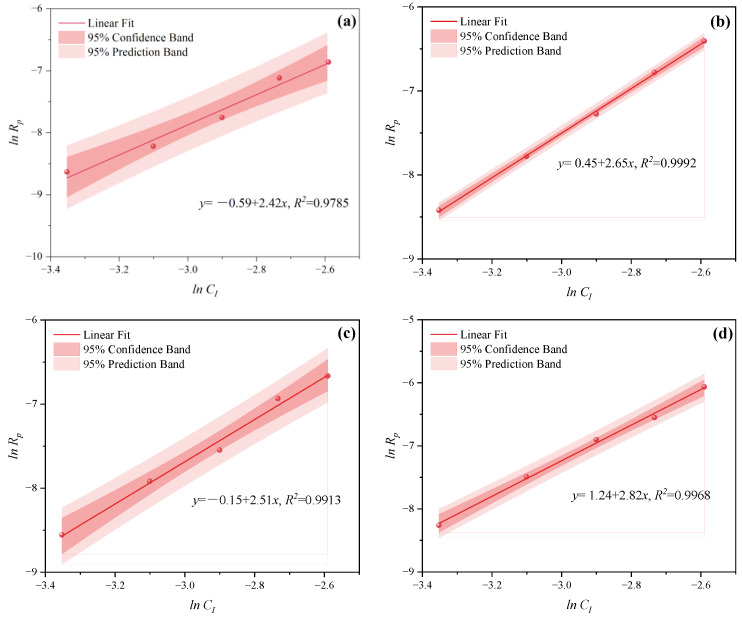
Effects of *C_I_* on *R_p_* at *n*_*H*_2_*O*_2__:*n_VC_* of (**a**) 3.5:1, (**b**) 4.65:1, (**c**) 5.85:1, and (**d**) 7:1 under CI.

In the free radical initiation system used for polymer synthesis, the initiator is instrumental in regulating the polymerization rate *R_p_*. As a common initiator, H_2_O_2_ can produce two hydroxyl radicals through thermal decomposition, but its decomposition activation energy is high. It is more effective when used in conjunction with a reducing agent. In this study, a dual initiator system of H_2_O_2_ and VC was utilized. The impacts of various *C_I_* on the polymerization kinetics of PCE were examined at *n*_*H*_2_*O*_2__:*n_VC_* = 3.5:1, 4.65:1, 5.85:1, and 7:1. The impacts of *C_I_* (0.035, 0.045, 0.055, 0.065, and 0.075 mol·L^−1^) were investigated while the other process parameters remained constant. The effects of *C_I_* on *C_V_* under MI and CI are shown in Figure 6 and Figure 7. The *C_V_* displayed an increasing trend with the increase of CI at *n*_*H*_2_*O*_2__:*n_VC_* =3.5:1, 4.65:1, 5.85:1, and 7:1. A higher *C_I_* led to the decomposition of more primary radicals, thereby enhancing the likelihood of collisions and improving initiation efficiency. The slopes of MI polymerization all exceeded 2.5 and were 3.030 in Figure 8a, 2.901 in Figure 8b, 2.848 in Figure 8c, and 2.655 in Figure 8d, which showed a good linear relationship between *ln C_I_* and *ln Rp*. Hence, under MI, the effects of *C_I_* on *Rp* can be expressed as *Rp∝C_I_*^3.030^, *Rp∝C_I_*^2.901^, *Rp∝C_I_*^2.848^, and *Rp∝C_I_*^2.655^, respectively. Under the CI method, the slopes were, respectively, 2.824 as shown in in Figure 9a, 2.650 in Figure 9b, 2.511 in Figure 9c, and 2.429 in Figure 9d, only above 2. Therefore, the effects of *C_I_* on *Rp* can be expressed as *Rp∝C_I_*^2.824^, *Rp∝C_I_*^2.650^, *Rp∝C_I_*^2.511^, and *Rp∝C_I_*^2.429^, respectively. Therefore, the most favorable CI and *n*_*H*_2_*O*_2__:*n_VC_* on *C_V_* and *R_p_* both were acquired at 0.075 mol·L^−1^ and 3.5:1 under two different heating methods.

**Figure 10:**
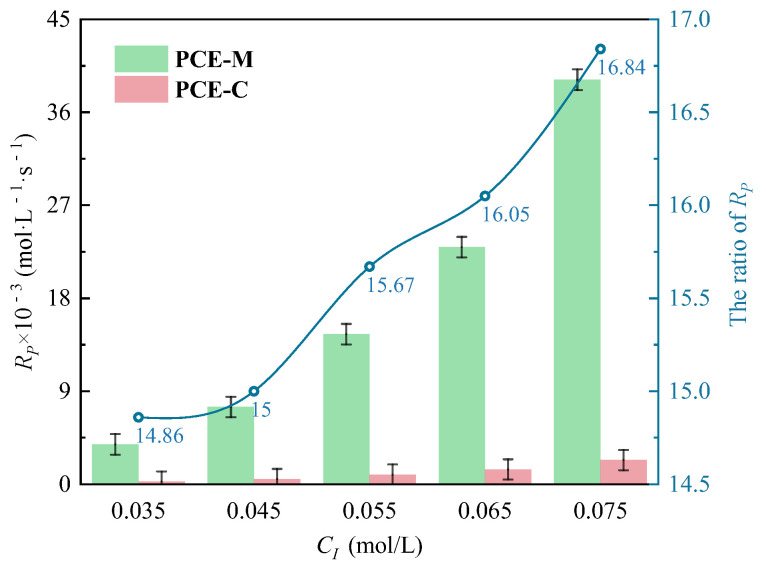
Comparison of *C_I_* on *R_p_* at *n*_*H*_2_*O*_2__:*n_VC_* of 3.5:1 under two different heating methods.

According to Figure 10, *Rp* under both MI and CI gradually increased with the increase of *C_I_*. Similarly, *Rp* under MI method exceeded that under the CI method at each *C_I_* (0.035, 0.045, 0.055, 0.065, and 0.075 mol·L^−1^), and *Rp* under MI was 14.86~16.84 times that under CI. 

In conclusion, the kinetic equations of two different heating methods can be written as:

(11) $$R_{P}\propto C_{M}^{4.157}\cdot C_{I}^{3.030}\;\;(microwave\;induction\;polymerization)$$

(12) $$R_{P}\propto C_{M}^{2.133}\cdot C_{I}^{2.824}\;(conventional\;thermal\;polymerization)$$

#### 3.1.3. The Effect of Power on C_V_ and R_p_

**Figure 11:**
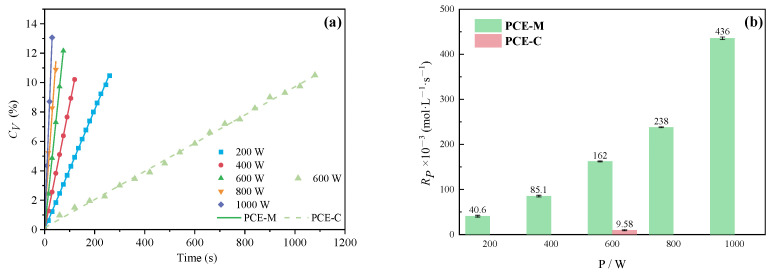
Effects of power on *C_V_* (**a**) and *Rp* (**b**).

Microwave power generally affects the heating rate of the reaction system, but applying too high microwave power to the reaction system for a long time will cause the reaction system to overheat. Therefore, an appropriate microwave power should be selected to heat the system at an appropriate heating rate. The effects of microwave power were evaluated at 200 W, 400 W, 600 W, 800 W, and 1000 W, and the remaining process parameters were constant. As shown in Figure 11, *C_V_* and *Rp* gradually increased with the gradual increase of microwave power. These results indicated that MI can significantly reduce reaction time and improve product conversion rates at equivalent power inputs, offering a pathway to greener and more energy-efficient PCE synthesis [33]. It can be found that MI can greatly reduce the reaction time and improve the product conversion rate under the same input power level. Therefore, microwave technology can achieve green and energy-saving preparation of PCE.

#### 3.1.4. The Effect of Polymerization Temperature on C_V_ and R_p_

**Figure 12:**
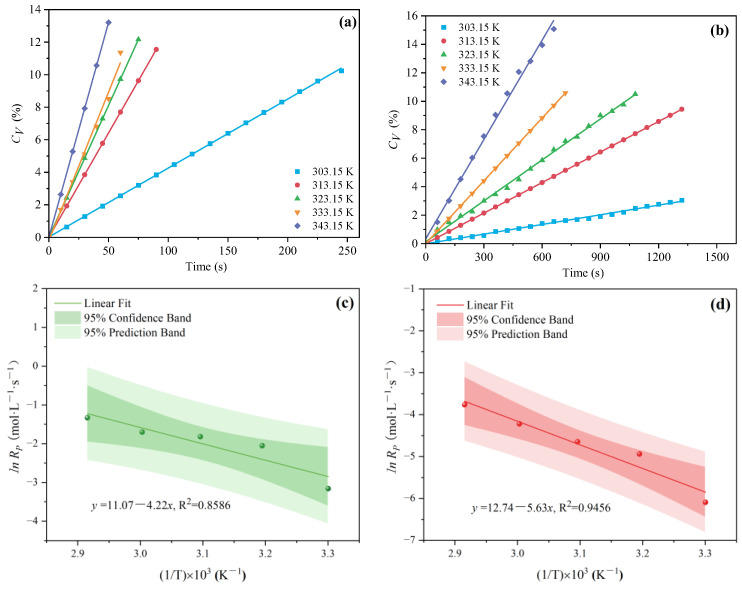
Effects of polymerization temperature on *C_V_* (**a**,**b**) and *Rp* (**c**,**d**) under two different heating methods.

**Figure 13:**
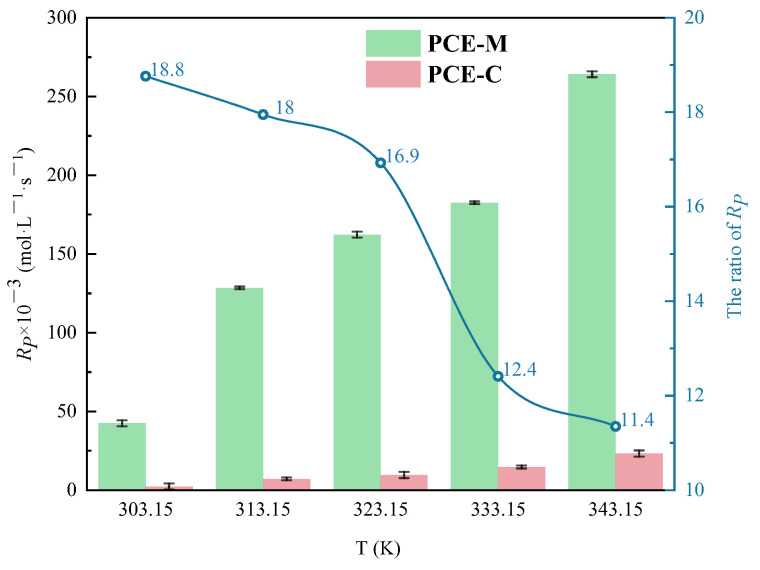
Comparison of polymerization temperature on *Rp* under two different heating methods.

In general, an increase in temperature generally accelerates the decomposition of the initiator, thereby increasing the polymerization rate. In this study, the effects of polymerization temperature (303.15, 313.15, 323.15, 333.15, and 343.15 K) were evaluated. As can be seen from Figure 12a,b, *C_V_* increased with increasing polymerization temperature under MI and CI. It was found that $lnR_{P}\;$and 1/T showed a linear relationship. As shown in Figure 12c,d, the slopes of MI and CI polymerization were −4.22 and −5.63, respectively. Compared with the CI method, applying microwave to the polymerization reaction system of PCE reduced the barrier of activation energy ($E_{a}$) (from 46.83 kJ·mol^−1^ to 35.07 kJ·mol^−1^), which can be calculated by Equation (6). Unlike other reaction conditions, the advantage of *Rp* under MI was weakened, and namely, the thermal effect of microwave was weakened with the increase of polymerization temperature. As shown in Figure 13, *Rp* under MI was 11.35~18.76 times that under CI. Even so, at a certain temperature, *Rp* under the two different heating methods were significantly different. This difference was attributed to the selective heating effect of polar molecules under MI, where the measured temperature in a microwave reaction did not equate to the actual reaction temperature [34]. Polar molecules absorb microwave energy and immediately convert it to heat, a process completed instantly upon heating, thereby circumventing the heat conduction delay typically encountered in reactions [35,36]. Consequently, this rapid energy conversion triggered a cascade of physical effects: increased reaction rates, reduced thermal energy loss, and swift temperature rise [37].

### 3.2. Mechanism Analysis of Accelerated R_p_ in PCE Polymerization System: Microwave Thermal Effect

When a substance is exposed to an electromagnetic field, its internal dielectric dissipation results in bulk or dielectric heating. Partially absorbed microwave energy is transformed into heat energy by electromagnetic induction material in the microwave field [34,35]. Ion conduction and dipole polarization are two common heating processes for compounds in a reaction system under the influence of a microwave electric field. According to the ion conduction process, charged ions oscillate back and forth in the microwave electromagnetic field, collide with nearby molecules, and produce heat because of the friction. The mechanism of dipole polarization is that, due to the unbalanced charge distribution in the molecules, polar molecules in the reaction system or non-polar molecules that can produce instantaneous dipoles oscillate rapidly at a rate of 4.91 times per second under the influence of microwave electric field components [38]. The vibration of molecular dipoles should closely match the vibration of the magnetic field because of the friction and dielectric loss between molecules. However, the molecular dipole’s oscillation frequently exhibits the hysteresis effect, which results in the energy being used up as heat, or microwave thermal effect. Since the dielectric properties of the reactants rely on their dipole moments, it is clear from the preceding that the microwave thermal effect is directly connected to those qualities. Due to the dipole moment and dielectric characteristics of the reactants, the acceleration mechanism of the polymerization rate in the PCE polymerization system was examined in this study.

**Figure 14:**
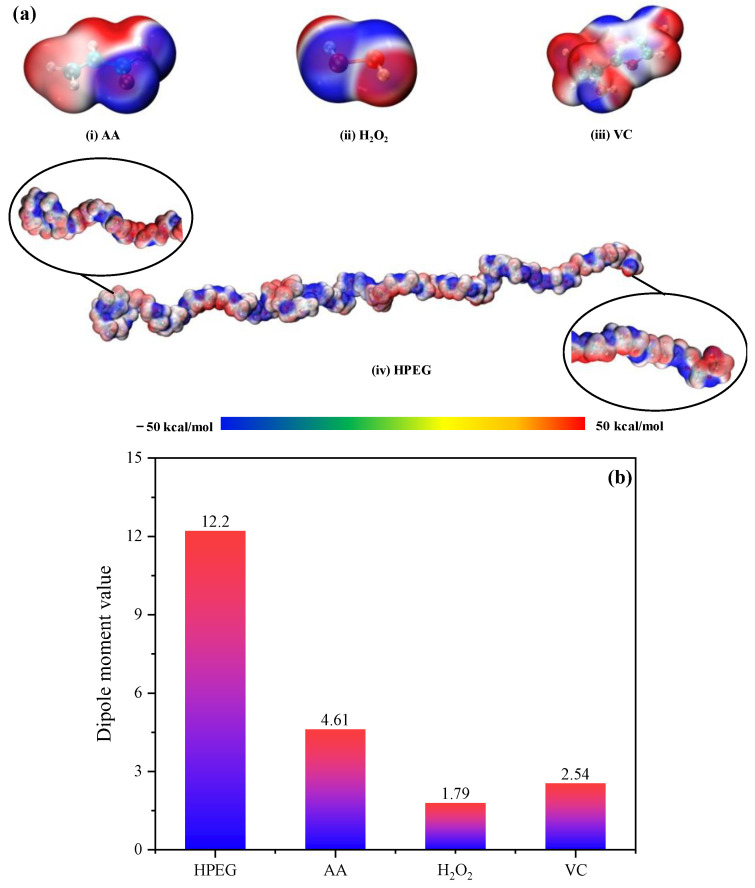
(**a**) ESP of main reactants, (i) AA, (ii) H_2_O_2_, (iii) VC, (iv) HPEG; (**b**) dipole moment of main reactants.

The electrostatic potential (ESP) and dipole moment of the main reactants in PCE polymerization system were calculated by density functional theory (DFT). To evaluate the reactivity of the four molecular surfaces, the ESP of the main reactants was first analyzed. The ESP surface is visualized by drawing different colors of electron density around the entire molecule. The color change describes information about the reaction potential. The ESP surfaces of the four molecules were displayed by Multiwfn software (3.8), as shown in Figure 14a. The blue of electron density indicates negative ESP, which represented the electrophilic region. The positive charge density was defined as the nucleophilic region, which was mapped red. White reflected the Van der Waals effect, corresponding to the zero value of the electrostatic potential. For AA and VC molecules, the electrophilic region was on the O atom and the nucleophilic region was around the H atom. Similar results were also applicable to H_2_O_2_ molecules, but the H site showed more obvious nucleophilic reactivity. This phenomenon may be due to the local neutral region caused by C atoms in AA and H_2_O_2_. The HPEG molecule showed a clear blue area, mixed with a partial light red area and a white area, indicating that HPEG may have a stronger ability to attract electrophiles. The dipole moment simulation results of the main reactants are shown in Figure 14b, and their dipole moment distribution ranges from 2 to 13. The dipole moment value in descending order was as follows: HPEG, AA, VC, H_2_O_2_. Therefore, the order of polarity of the four molecules from strong to weak was HPEG, AA, VC, H_2_O_2_. In addition, compared with AA, the polarity of HPEG was higher, and it can vibrate at higher frequencies under microwave field, which increased the collision probability of the molecule to other molecules. In this study, the reason why HPEG had higher polymerization activity than AA in the microwave field was explained essentially from the perspective of molecular dielectric properties. This was also the fundamental reason that HPEG had a higher reactivity than AA in Reference [21].

**Figure 15:**
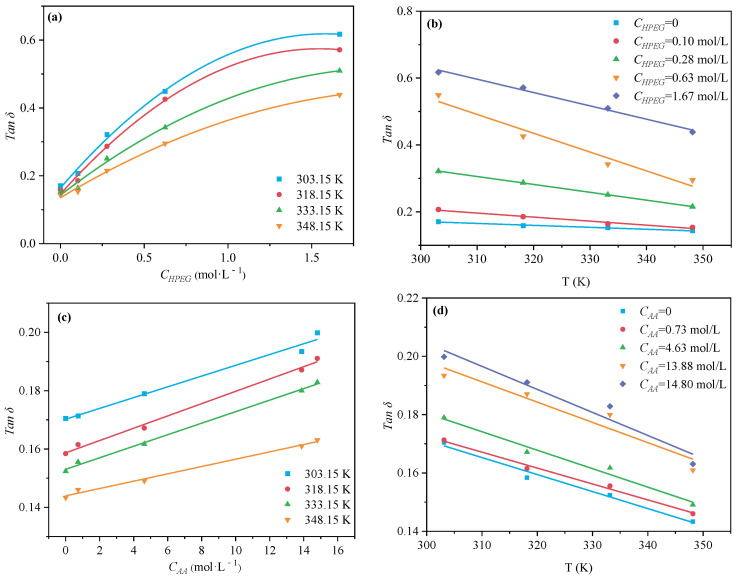
Effects of different monomer concentrations and temperatures on tan δ of HPEG (**a**,**b**) and AA (**c**,**d**).

**Figure 16:**
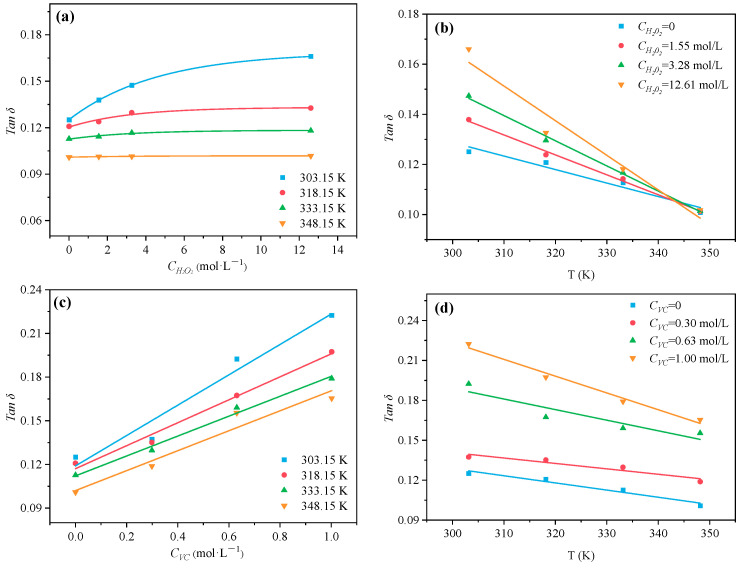
Effects of different initiator concentrations and temperatures on tan δ of H_2_O_2_ (**a**,**b**) and VC (**c**,**d**).

Loss tangent (tanδ) is used to characterize the capacity of a reactant to change over electromagnetic energy into thermal energy at a given microwave frequency and temperature. A reaction medium with a high tanδ value can absorb microwave energy more effectively and be heated rapidly in the microwave field. Kumar [39] divides substances into three classes according to tanδ: (i) high (tanδ > 0.5), good microwave absorbency and facilitating efficient heating; (ii) middle (tanδ ≈ 0.1–0.5), medium microwave absorbency; and (iii) very low (tanδ < 0), very poor microwave absorbency. In this experiment, the interaction between electromagnetic wave and the reactants of the system are studied by tanδ. As can be seen from Figure 15, tanδ of HPEG and AA were both positively associated with its concentration at a certain temperature. Namely, the inclusion of HPEG and AA improved the mixed solution’s ability to convert microwave electromagnetic energy into heat. Comparing Figure 15a,c, the concentration of HPEG required for the same tanδ value was significantly smaller than that of AA, which also meant that the effect of HPEG on tanδ was greater than that of AA on tanδ. When the concentration of HPEG or AA was constant, tanδ had a negative correlation with temperature (Figure 15b,d); namely, the ability of the solution to convert microwave electromagnetic energy into heat energy decreases. In other words, the high temperature was not conducive to the thermal effect of the mixed solution. Similarly, the addition of H_2_O_2_ or VC enhanced microwave energy conversion at lower temperatures, with VC exhibiting better microwave absorption than H_2_O_2_, as indicated in Figure 16.

**Figure 17:**
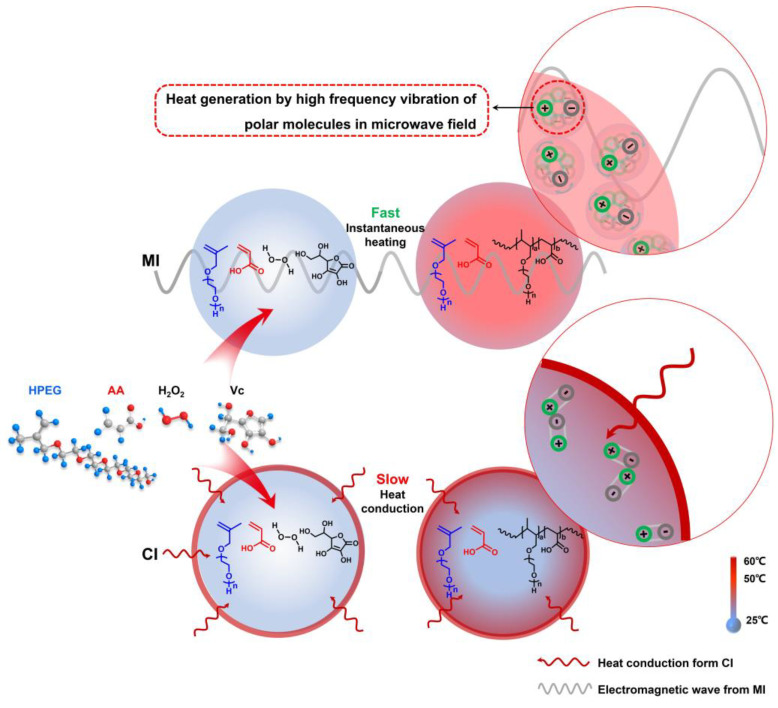
Mechanism of MI and CI on polymerization rate and conversion in the reaction system.

The above dielectric characteristic parameters and the corresponding mechanism were analyzed in the conclusions of Section 3.1.1 and Section 3.1.2. Because HPEG, AA, H_2_O_2_, VC, and solvent water are polar molecules in the PCE synthesis system, the charge distribution in these polar molecules is unbalanced under the microwave field. Based on this, molecules oscillate at high frequency, resulting in friction and dielectric loss within or between molecules. In this process, the polar molecule instantly becomes a miniature “molecular heater”, that is, the molecule absorbs microwave energy and instantly converts it into heat (see Figure 17). Therefore, the microwave energy can uniformly flow through the whole heated material, realizing rapid and uniform internal heating, avoiding the temperature gradient that occurs in the conventional thermal induction reaction system, and greatly improving the heating efficiency. The unique heating mechanism of microwaves greatly increases the probability of intermolecular collision. Thus, the *R_p_* of the polymerization system was improved, and the conversion rate of the reaction was also greatly improved.

## 4. Conclusions

In summary, PCEs were prepared by monomers (AA and HPEG) and initiators (H_2_O_2_ and VC) through free radical polymerization. The polymerization kinetics of the MI and CI methods were compared and discussed. The findings of the experimental studies were as follows:

(1) Under MI, the influence of total monomer concentration on the polymerization rate *Rp* was quantified by proportional relationships *Rp∝C_M_*^4.157^, *Rp∝C_M_*^4.024^, *Rp∝C_M_*^3.928^, and *Rp∝C_M_*^3.816^ at *n_AA_*:*n_HPEG_* = 2.5:1, 3:1, 4:1, and 5:1, respectively. Furthermore, the initiator concentration effects of *Rp∝C_I_*^3.030^, *Rp∝C_I_*^2.901^, *Rp∝C_I_*^2.848^, and *Rp∝C_I_*^2.655^ were also established at *n*_*H*_2_*O*_2__:*n_VC_* = 3.5:1, 4.65:1, 5.85:1, and 7:1, respectively.
(2) Under CI, the total monomer concentration effects on *Rp* were depicted as *Rp∝C_M_*^1.856^, *Rp∝C_M_*^1.916^, *Rp∝C_M_*^2.088^, and *Rp∝C_M_*^2.133^ at *n_AA_*:*n_HPEG_* = 2.5:1, 3:1, 4:1, and 5:1, respectively. In addition, the initiator concentration effects of *Rp∝C_I_*^2.824^, *Rp∝C_I_*^2.650^, *Rp∝C_I_*^2.511^, and *Rp∝C_I_*^2.429^ were also established at *n*_H_2_O_2__:*n_VC_* = 3.5:1, 4.65:1, 5.85:1, and 7:1, respectively.
(3) In polymerization processes, microwaves can enhance reaction rates and shorten reaction time. Compared with the CI method, applying microwave to the polymerization reaction system of PCE reduced the barrier of activation energy from 46.83 kJ·mol^−1^ to 35.07 kJ·mol^−1^.
(4) The principal reactants in polymerization were polar molecules that absorbed microwave energy and quickly converted it to heat energy. The temperature gradient that occurs in in the typical heat conduction response system was avoided by this heating mechanism, which considerably enhanced heating efficiency. At the same time, the chance of intermolecular collisions was considerably raised, which improved the polymerization system *Rp* but also greatly improved the reaction’s conversion rate.

## Data Availability

Data are contained within the article.
